# Incidentally detected massive scrotal cystocele

**DOI:** 10.1590/0100-3984.2016.0145

**Published:** 2018

**Authors:** Felipe Welter Langer, Giordano Rafael Tronco Alves, Gustavo Suertegaray, Daiane Santos, Carlos Jesus Pereira Haygert

**Affiliations:** 1 Universidade Federal de Santa Maria (UFSM) - Radiologia e Diagnóstico por Imagem, Santa Maria, RS, Brazil

**Figure 1:**
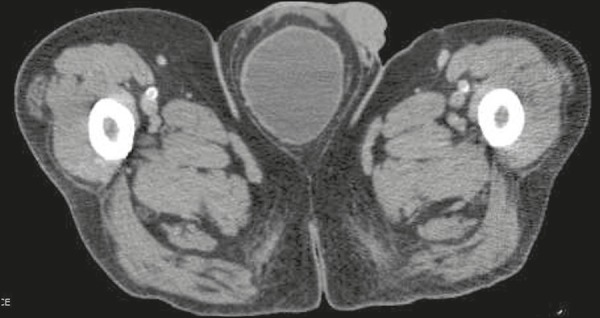
Axial CT scan of the pelvis showing the urinary bladder herniated into the scrotum.

**Figure 2:**
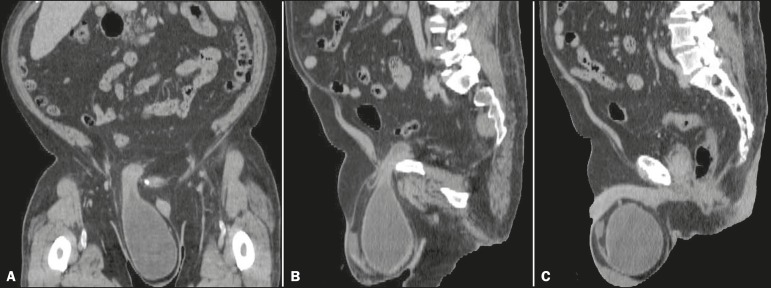
CT images of the abdomen and pelvis, in a coronal view (A) and in sagittal views (B,C), showing a massive right-sided scrotal cystocele.

Dear Editor,

A 65-year-old male patient was referred to our institution for investigation of a 10-year history of epigastric pain. His pain had been progressively worsening during the past months, intensified after the consumption of solid foods. The only notable aspect of his medical history was arterial hypertension. He complained of nocturia, awakening to void about six times per night, but did not report dysuria, hematuria, scrotal swelling, or other urinary tract symptoms. Physical examination revealed epigastric tenderness and hepatosplenomegaly. Upper gastrointestinal endoscopy showed an ulcerated lesion on the greater curvature of the stomach. In the analysis of the biopsy sample, the lesion was classified as non-Hodgkin lymphoma. A computed tomography (CT) scan of the abdomen and pelvis, performed for staging, revealed an unsuspected massive inguinoscrotal hernia of the urinary bladder, a condition known as scrotal cystocele (Figures 1 and 2). The CT scan also showed moderate right-sided uronephrosis, which was attributed to extrinsic compression of the right ureter. The results of the urinalysis were unremarkable. After work-up of the non-Hodgkin lymphoma, a hernia reduction followed by inguinal herniorrhaphy was planned in order to prevent long-term complications of bladder herniation.

Although the urinary bladder is involved in up to 4% of inguinal hernias, massive scrotal cystocele is quite uncommon^(1)^. Advanced age, obesity, and male gender are recognized risk factors for bladder herniation^(2)^. Bladder herniation is usually asymptomatic, although some patients complain of voiding-related scrotal swelling, two-stage micturition (a first spontaneous voiding followed by a second requiring manual compression of the inguinoscrotal region), urinary tract infections, or irritative lower urinary tract symptoms (LUTS) such as urgency, frequency, and nocturia secondary to bladder outlet obstruction or infection^(1-3)^. Possible complications of untreated scrotal cystocele include hydronephrosis, renal failure, cystolithiasis, vesicoureteral reflux, bladder necrosis, and bladder perforation^(2,4,5)^.

The preoperative diagnosis of scrotal cystocele is important to prevent iatrogenic injury of the herniated bladder during repair surgery^(3)^. The condition should be suspected in all patients presenting with inguinal hernias and concomitant renal failure or LUTS, especially if a painless unilateral scrotal swelling is detected^(4,5)^. However, as illustrated by our case, the absence of clinically detectable scrotal swelling should not exclude the hypothesis of bladder herniation, nor should it preclude further investigation. The imaging diagnosis can be established by CT, ultrasound, cystography, or intravenous pyelography^(2)^. Because CT provides a clear anatomical outline of the herniated contents and allows prompt identification of complications, thereby enabling appropriate surgical planning, it is an especially valuable tool in the work-up of scrotal cystocele^(6)^.

Hernia repair has shown to be effective in improving LUTS and reducing complications in patients with significant bladder herniation; therefore, standard treatment of scrotal cystocele consists of reduction or resection followed by herniorrhaphy^(1)^. Acute bladder infarction or urinary obstruction can require urgent laparotomy with resection of the affected portion of the bladder^(3)^. In the elective setting, partial bladder resection is often restricted to patients presenting with bladder necrosis, a tumor in the herniated bladder, or a bladder diverticulum^(2)^. However, timely preoperative diagnosis of scrotal cystocele remains the single most important determinant of a successful surgical outcome, making proper clinical and imaging assessments invaluable.
